# Cloning, Expression, and Structural Elucidation of a Biotechnologically Potential Alkaline Serine Protease From a Newly Isolated Haloalkaliphilic *Bacillus lehensis* JO-26

**DOI:** 10.3389/fmicb.2020.00941

**Published:** 2020-06-03

**Authors:** Hitarth B. Bhatt, Satya P. Singh

**Affiliations:** UGC-CAS Department of Biosciences, Saurashtra University, Rajkot, India

**Keywords:** recombinant alkaline protease, gene expression, Little Rann of Kutch, structure–function relationship, detergent additive, whey protein hydrolysis

## Abstract

An alkaline protease gene of *Bacillus lehensis* JO-26 from saline desert, Little Rann of Kutch, was cloned and expressed in *Escherichia coli* BL21 (DE3). A 1,014-bp ORF encoded 337 amino acids. The recombinant protease (APrBL) with Asp 97, His 127, and Ser 280 forming catalytic triad belongs to the subtilase S8 protease family. The gene was optimally expressed in soluble fraction with 0.2 mM isopropyl β-D-thiogalactopyranoside (IPTG), 2% (w/v) NaCl at 28°C. APrBL, a monomer with a molecular mass of 34.6 kDa was active over pH 8–11 and 30°C−70°C, optimally at pH 10 and 50°C. The enzyme was highly thermostable and retained 73% of the residual activity at 80°C up to 3 h. It was significantly stimulated by sodium dodecyl sulfate (SDS), Ca^2+^, chloroform, toluene, n-butanol, and benzene while completely inhibited by phenylmethylsulfonyl fluoride (PMSF) and Hg^2+^. The serine nature of the protease was confirmed by its strong inhibition by PMSF. The APrBL gene was phylogenetically close to alkaline elastase YaB (P20724) and was distinct from the well-known commercial proteases subtilisin Carlsberg (CAB56500) and subtilisin BPN′ (P00782). The structural elucidation revealed 31.75% α-helices, 22.55% β-strands, and 45.70% coils. Although high glycine and fewer proline residues are a characteristic feature of the cold-adapted enzymes, the similar observation in thermally active APrBL suggests that this feature cannot be solely responsible for thermo/cold adaptation. The APrBL protease was highly effective as a detergent additive and in whey protein hydrolysis.

## Introduction

Proteases, being among the most important groups of the industrial enzymes, represent 60% of the total enzyme market (Jaouadi et al., 2008). The demand has increased due to its applications in various sectors such as detergent, leather, food, dairy, pharmaceutical, textile, and peptide synthesis (Haki and Rakshit, 2003; Jain et al., 2012; Purohit and Singh, 2013; Sinha and Khare, 2013; Raval et al., 2014).

Majority of the industrial processes are carried out under the extreme conditions of temperature, pH, and high concentrations of organic solvents, where majority of the enzymes fail to function. Therefore, enzymes of haloalkaliphile origin will serve the purpose of stability and activity at multiple of extremities (Raval et al., 2018). Proteases from halophilic microorganisms are in demand for various applications due to their ability to function at alkaline pH, high temperatures, and high salt concentrations (Sinha and Khare, 2013; Raval et al., 2014). Thus, they are also suitable for food processing under saline conditions. Proteases are widely used in detergents due to inefficiency of non-enzymatic detergents for the removal of protein-containing materials from the textile fibers. The enzymes not only enhance the washing efficiency but also shorten the washing duration at moderate temperatures.

Whey contains approximately 20% of the total soluble milk proteins (Walstra and Jenness, 1984). In protein hydrolysis, peptide bonds are cleaved, resulting in peptides of different sizes and free amino acids. Protein hydrolysis is carried out either chemically or enzymatically. Chemical methods usually yield products with modified amino acids and reduced nutritional values. Therefore, the enzymatic hydrolysis is performed under moderate conditions without side reactions and loss of nutritional values (Tavano, 2013).

*Bacillus* strains are suitable sources of the commercial enzymes due to their ability to secrete enzymes in a short time (Maurer, 2004). As a matter of fact, different species of *Bacillus* genus are among the most common sources of commercial proteases (Contesini et al., 2018). Although many *Bacillus* strains produce alkaline proteases (Patel et al., 2006; Dodia et al., 2008; Jain et al., 2012; Raval et al., 2014), we need enzymes with better efficiency and features to perform under stressful conditions. Therefore, the search for suitable enzymes continues for various applications. Alkaline proteases are reported from bacteria and actinomycetes of different marine habitats (Thumar and Singh, 2009; Gohel and Singh, 2012, 2018; Annamalai et al., 2014; Raval et al., 2014, 2015; Thakrar and Singh, 2019; Sharma et al., 2020). However, only limited studies are available on the microorganisms of desert origin. The Little Rann of Kutch is a saline desert largely unexplored for its microbial diversity and biotechnological potential (Bhatt and Singh, 2016; Bhatt et al., 2017, 2018).

Further, a high level of production is desirable for the commercialization of any enzyme from microbial sources. In view of the investigations on the gene expression and cost-effectiveness of the enzymes, the protease gene was cloned and expressed in mesophilic host, *Escherichia coli*. Only few reports exist on the gene cloning and characterization of the recombinant proteases of haloalkaliphilic microorganisms. Besides, sequence information of the protease gene will help in elucidating the structure–function relationship and identifying various functional regions of the encoded enzymes.

In consideration of the above scenario, we cloned an alkaline protease (APrBL) gene from a newly isolated haloalkaliphilic *Bacillus lehensis* and expressed into *E. coli* BL21 (DE3), a mesophilic host. The gene expression was optimized with respect to induction, growth temperature, and NaCl concentrations. We purified and characterized the recombinant protease and assessed its potential as detergent additive and in whey protein hydrolysis. The study established the potential of this serine alkaline protease in detergent and food industries. This study represents the first account on the gene cloning, analysis of the expression, and characterization of a recombinant protease from a haloalkaliphilic bacterium of the saline desert, Little Rann of Kutch.

## Materials and Methods

### Bacterial Strains and Vectors

A haloalkaliphilic bacterium *B. lehensis* JO-26 was isolated from the saline desert of Little Rann of Kutch (India). *E. coli* BL21 (DE3) (Merck Millipore, Germany) was used for the expression studies and was grown in LB supplemented with kanamycin (50 μg/ml) for the recombinant protein expression. pET28a (+) (Novagen, CA, United States) was used as an expression vector. Plasmid preparation and DNA purification were carried out using commercial kits (Qiagen, Germany). Restriction enzymes and other molecular biology reagents were from commercial sources (Roche, Germany).

### Genomic DNA Extraction, Primer Designing, and Gene Amplification

Genomic DNA was extracted from 2 ml of activated growth cultures (OD600 ∼0.8) using the DNeasy Blood & Tissue Kit for DNA (Qiagen, Germany). Isolated DNA was used as a template for the amplification of the alkaline protease (APrBL) gene. ORF finder tool of NCBI was used to identify the open reading frame of the APrBL gene. Based on the whole genome sequence of *B. lehensis* available in the NCBI database, a set of primer pair (APrBL *Nde*I F′ CCG**CATATG**GCGCAGGTTGGAACATTTTG and APrBL *Xho*I R′ CCG**CTCGAG**CGAGTAGGTCTCTTTTGCAG) was designed to obtain the APrBL gene without secretion signal. Restriction sites are underlined and highlighted in bold. Using APrBL F & R primers, the APrBL gene was amplified using Emerald green master mix (Takara, Japan) under the optimized PCR program (denaturation step at 95°C for 5 min, followed by 35 cycles of denaturation at 95°C for 50 s, annealing at 56°C for 45 s and extension at 72°C for 1 min 30 s with final extension at 72°C for 7 min) in 25-μl reaction mixture using a thermocycler (Eppendorf, Germany).

### Construction of the Recombinant APrBL-pET28a

A set of primers APrBL Forward and Reverse with *Nde*I and *Xho*I restriction sites was used to amplify the APrBL gene. The amplified product APrBL (200 ng) and expression vector pET28a (2 μg) were digested with *Nde*I and *Xho*I restriction enzymes as per the manufacturer’s instructions. The digested amplified product and vector were purified using the HiPurA PCR product purification kit (Himedia, India). Double-digested vector pET28a (+) (133 ng) and APrBL (88 ng) were ligated using T4 DNA ligase (Roche, Germany) at 16°C overnight. The ligated product was transformed into competent *E. coli* BL21 (DE3) cells. Colony PCR and double digestion of the plasmids with *Nde*I and *Xho*I were carried out to confirm the positive APrBL-pET28a clones.

### Gene Expression: Effect of Isopropyl β-D-Thiogalactopyranoside, Temperature, and Salt

Seed culture of *E. coli* BL21 harboring APrBL-pET28a vector was prepared by growing cells on a rotary shaker (200 rpm) for overnight at 37°C. LB medium (50 ml) containing kanamycin (50 μg/ml) was inoculated with 1% (v/v) inoculum and incubated until optical density reaches to 0.5–0.6 at 600 nm. It was then followed by the addition of isopropyl β-D-thiogalactopyranoside (inducer, IPTG) and incubation for 20 h on a rotary shaker (200 rpm). Protease expression was examined at the IPTG concentrations of 0.2 and 1 mM and at 28 and 37°C to optimize the distribution of the expressed protease in soluble fraction. The cells were harvested at 14,031 × g (Beckman Coulter Allegra 64R Centrifuge, United States) for 10 min at 4°C, and the cell pellet was washed with buffer containing 50 mM NaH_2_PO_4_ and 100 mM NaCl (pH 8). The cells were disrupted by sonication for 30 s in six cycles with Sartorius Labsonic M at 30 Hz. Between each cycle, the samples were chilled in ice for 30 s. The resulting supernatant was considered as soluble fraction. The pellets were treated with 8 M urea for 30 min at 30°C, followed by centrifugation at 9,744 × *g* for 5 min at 4°C to obtain supernatant considered as the insoluble fraction (Purohit and Singh, 2013). Insoluble and soluble fractions of the induced and uninduced cells were analyzed by sodium dodecyl sulfate (SDS)–polyacrylamide gel electrophoresis (PAGE) on a 12% polyacrylamide gel. Similarly, the effect of NaCl concentrations (0–4%, w/v) was examined on the expression of protease and its distribution in soluble fraction with other optimum conditions of 0.2 mM IPTG and 28°C growth temperature. The expression was analyzed by plate diffusion assay and enzyme assay as described below.

### *In silico* Analysis of APrBL

Nucleotide sequence of APrBL gene was used for phylogenetic analysis and was compared against NCBI database using BLASTn tool. Similarly, the protein sequence of APrBL was compared against the protein database using BLASTp tool. Multiple sequence alignment (MSA) was achieved using Clustal W software. The phylogenetic tree was constructed using the Neighbor-Joining method of MEGA 6 software (Tamura et al., 2013). Distances were calculated using the Kimura correction in a pairwise deletion manner (Kimura, 1980). The N-terminal signal peptide analysis was carried out by the SignalP version 5.0 program (Armenteros et al., 2019). The hydropathicity of the protein was determined using the Kyte and Doolittle scale (Kyte and Doolittle, 1982). Primary and secondary structure properties of the protease and amino acids were studied by Expasy and NCBI tools. Expasy’s Protparam server^1^ was used for detection of the physicochemical properties of these proteins. The number of amino acids, theoretical isoelectric point (p*I*), molecular weight, aliphatic index, instability index, grand average hydropathy (GRAVY), and total number of positive and negative residues were computed for each protein. Expasy’s PROSITE tool^2^ was used to determine the catalytic triad of the enzyme. The secondary structure configuration of APrBL was predicted by Endscript 2.0 (Gouet et al., 2003) and SOPMA tool (Geourjon and Deleage, 1995). Three-dimensional structure of the serine protease was modeled using the I-TASSER server for three-dimensional (3D) structure prediction and validation (Yang et al., 2015). The model was also validated by structure assessment tool of SWISS-MODEL (Kiefer et al., 2009). Further, the hydropathy profile was plotted according to the method of Kyte and Doolittle using pscale tool (Kyte and Doolittle, 1982).

### Homology Modeling

APrBL amino acid sequence without signal peptide was used for homology modeling. SWISS-MODEL Workspace server was used to identify the template and building protein model^3^ (Kiefer et al., 2009).

### Purification of the Recombinant Alkaline Protease

The recombinant protease (APrBL) was purified under non-denaturing conditions using Novagen Ni-NTA His⋅Bind resin. The IPTG-induced recombinant *E. coli* BL21 (DE3) cells were sonicated in binding buffer containing 50 mM phosphate buffer (pH 8), 100 mM NaCl, 10 mM MgCl_2_, 1 mg ml^1^ lysozyme, 5 mM β-mercaptoethanol (β-ME), and 5% (v/v) glycerol. After sonication, debris was spun down and the supernatant containing APrBL was collected for purification. The chromatographic column was packed with Ni-NTA His⋅Bind Resin (Merck) and allowed to settle to generate 2 ml of bed volume. The packed Ni-NTA resin was washed with a 5-column volume of the deionized water to remove the ethanol. The column was then equilibrated with a 5-column volume of the binding buffer. Thereafter, the supernatant was passed through the column several times for binding of the 6 × histidine-containing recombinant protein to the Ni-NTA resin. After binding, 2-column volume of wash buffer (binding buffer + 20 mM imidazole) was passed through the column in order to wash out non-specific proteins and other impurities. The recombinant protein was eluted with one column volume of the elution buffer (binding buffer + imidazole) with 250 mM and 500 mM of imidazole in increasing gradient, and fractions were collected. The fractions were examined for purity of the enzyme on 12% SDS–PAGE. The protease activity of the purified APrBL was confirmed by enzyme assay.

### Protease Assay and Protein Estimation

The protease activity was measured by Anson–Hagihara’s method using tyrosine as standard (0–100 g/ml) (Hagihara, 1958). The enzyme (0.5 ml appropriately diluted enzyme) was added to 3.0 ml casein [0.6% (w/v) in 20 mM NaOH–Borax buffer, pH 10], and the reaction mixture was incubated at 50°C for 10 min. The reaction was terminated by the addition of 3.2 ml of TCA mixture (0.11 M trichloro acetic acid, 0.22 M sodium acetate, and 0.33 M acetic acid) and incubated at room temperature for 30 min. The precipitates were removed by filtration through Whatman-1 filter paper, and the absorbance of the filtrate was measured at 280 nm. One unit of alkaline protease activity was defined as the amount of enzyme liberating 1 μg of tyrosine per minute under the assay conditions. Protein concentrations were determined by the method of Bradford (1976) using bovine serum albumin as a standard.

### Kinetic Parameters of APrBL

*V*_max_ and *K*_m_ were determined using Lineweaver–Burk double reciprocal (1/V versus 1/S) plot by measuring the activity at various concentrations of casein substrate (0.25–10 mg/ml) under the standard conditions. The value of the turnover number (K_cat_) was calculated using the following equation:

$$K_{\text{cat}}=\frac{V_{\text{max}}}{\left[E\,t\right]}$$

where [*E*] refers to the active enzyme concentration, *V*_max_ refers to the maximum reaction rate, and *K*_cat_ is defined as the maximum number of chemical conversions of substrate molecules per second that a single catalytic site will execute for a given enzyme concentration (Mechri et al., 2017). For the determination of *K*_cat_ and *K*_cat_/*K*_m_, the value of *V*_max_ was expressed in terms of μmoles/ml/min.

### Enzyme Secretion by Plate Diffusion Assay

The soluble fractions of the expressed enzyme were analyzed for the enzyme activity using plate diffusion assay. The enzyme preparations were dispensed into wells created in the gelatin agar plate (3% Agar + 1% gelatin in 50 mM glycine-NaOH buffer, pH 10). The plates were then incubated for 24 h at 50°C followed by the observation of the zone of gelatin hydrolysis around the wells.

### Biochemical Characterization of APrBL

#### Effect of Temperature and pH on the Activity and Stability of the Enzyme

The temperature profile of the enzyme was determined using standard assay at varying temperatures in the range of 30–80°C. For thermal stability, the enzyme was preincubated for 30 min at the temperatures in the range of 40–80°C. Aliquots were withdrawn at 1, 3, and 24 h, and the protease activity was determined. The residual activities were determined by taking the activity prior to incubation as 100%. The assay was performed in 20 mM borax-NaOH buffer (pH 10) using 0.6% (w/v) casein as a substrate.

The effect of pH on the enzyme activity was determined in buffers of different pH. To determine the optimum pH, sodium phosphate buffer (pH 7.0), Tris–HCl buffer (pH 8–9), borax-NaOH buffer (pH 10), and glycine-NaOH buffer (pH 11–12) were used. The pH stability was determined by preincubating the enzyme at pH 8–12. Aliquots were withdrawn at 1, 3, and 24 h, and the protease activities were determined.

#### Effect of Metal Ions on the Enzyme Activity

The effect of various metal ions on APrBL activity was assessed by preincubating the enzyme with 5 mM concentration of different metal ions: CaCl_2_, MgSO_4_, HgCl_2_, CoCl_2_, MnCl_2_, FeSO_4_, and ZnCl_2_ for 30 min. The residual activities were measured using casein as substrate at pH 10 and 50°C. The activity measured without metal ions was considered as 100%.

#### Effect of Solvents on the Enzyme Activity

The effect of organic solvents on APrBL activity was examined by preincubating the enzyme with 10% (v/v) of n-hexane, n-butanol, iso-propanol, ethanol, methanol, chloroform, glycerol, benzene, and toluene for 1 h. The residual activities were measured using casein as substrate at pH 10 and 50°C. For the control, the organic solvent was replaced with an equivalent amount of borax-NaOH buffer (20 mM, pH 10). The relative activities were calculated taking the activity of the control as 100%.

#### Effect of Inhibitors and Surfactants on Enzyme Activity

To evaluate the effects of inhibitors and surfactants on the enzyme activity, APrBL was preincubated with 5 mM of different inhibitors such as ethylenediaminetetraacetic acid (EDTA), β-ME, phenylmethylsulfonyl fluoride (PMSF), dithiothreitol (DTT), and 1% (w/v) of surfactants such as SDS, Triton X-100, and Tween 80 for 30 min at room temperature before the residual activities were measured. The activity without any additive was considered as 100%.

### Applications of the Recombinant Alkaline Protease APrBL

#### Detergent Additive: Wash Performance Analysis

Application of the recombinant alkaline protease APrBL was assessed as detergent additive by wash performance analysis. New cotton cloth pieces (6 cm × 6 cm) were stained with human blood (0.5 ml). Five different commercial detergents Ariel, Tide, Surf, Nirma, and Wheel were used at the concentration of 7 mg/ml to simulate washing conditions. Endogenous protease present in these detergents was inactivated by incubating the solid detergents at 70°C for 1 h prior to the addition of APrBL enzyme. The following sets were prepared:

1. Flask with tap water (25 ml).
2. Five flask with tap water (25 ml) + Each different detergent.
3. Five flask with tap water (25 ml) + Each different boiled detergent + APrBL (200 U).
4. Flask with tap water (25 ml) + APrBL (200 U).

Then, the stained cloth pieces were shake-incubated (100 rpm) in abovementioned flasks with different wash conditions at 40°C for 10 min containing a total volume of 25 ml of tap water. After incubation, cloth pieces were taken out, rinsed with water, and dried. Untreated stained cloth pieces and treated with only tap water were taken as control. The performance of the recombinant alkaline protease in bloodstain removal was evaluated by visual examination.

#### Whey Protein Hydrolysis

Whey solution was adjusted to pH 10 with 3 M NaOH solution. APrBL at 300 U was added into the whey solutions. Two negative controls were included to compare the effect of APrBL for whey protein hydrolysis. In one control, enzyme was replaced with equal amounts of borax-NaOH buffer (20 mM, pH 10), while in another control, denatured alkaline protease APrBL was added. The reaction mixture was incubated for 8 h at 45°C with continuous shaking. The aliquots were withdrawn at regular time intervals, and hydrolysis reactions were terminated by heating at 90°C for 15 min in water bath (Shu et al., 2018). Total protein content of these sets was determined before and after hydrolysis using Bradford’s method (Bradford, 1976).

Degree of hydrolysis (DH) was calculated using soluble protein content method (Morais et al., 2013; Sinha and Khare, 2015). One milliliter of 10% (w/v) trichloroacetic acid was added to 1 ml of the aliquot collected after enzymatic hydrolysis and incubated at 37°C for 30 min. The mixture was then centrifuged at 10,000 rpm for 10 min at room temperature, and the total content of the soluble protein was determined by Bradford’s method (Bradford, 1976). The DH was calculated as follows:

DegreeofHydrolysis(%)

  =Soluble⁢protein⁢content⁢in⁢ 10%⁢TCA⁢(mg)×100Total⁢protein⁢content⁢(mg)

## Results and Discussion

### Gene Amplification, Sequencing, and Phylogenetic Analysis

The APrBL gene without the signal sequence was amplified using primer pair (APrBL *Nde*I F/*Xho*I R), designed based on whole genome sequence of *B. lehensis* G1 (CP003923) available in NCBI database. Signal peptides usually have no role except the export of protein in the periplasmic space. Therefore, production of recombinant protein without signal peptide in *E. coli* would not alter its biochemical characteristics and function. Moreover, removal of signal peptide has been reported to increase the expression of recombinant protein (Gopal and Kumar, 2013). Some reports describe the expression of proteases without signal peptide (Joshi and Satyanarayana, 2013; Cheng et al., 2015; Devi et al., 2016).

The APrBL gene coding full-length ORF of 1,014 bp with a start and stop codon consisted of 337 amino acid residues: a pro-peptide of 71 and a mature protein of 266 amino acids. The molecular mass was 34.5 kDa, and isoelectric point (pI) was 4.66. The corresponding molecular weights with pro-peptide regions are reported earlier (Jaouadi et al., 2008; Deng et al., 2011; Cheng et al., 2015; Zhang et al., 2016). PROSITE analysis of the ORF revealed the catalytic triads of APrBL to be Asp 97, His 127, and Ser 280, a common feature of the serine proteases of subtilase superfamily (Siezen and Leunissen, 1997; Purohit and Singh, 2014).

**FIGURE 1:**
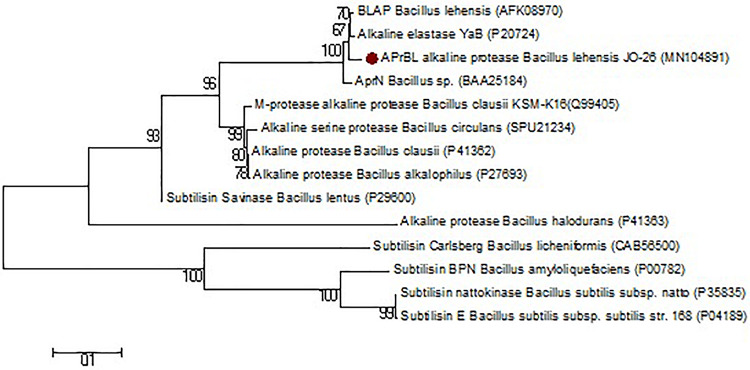
Phylogenetic tree based on a comparison of the APrBL amino acid sequence and some of their closest phylogenetic relatives retrieved from the NCBI database. The tree was reconstructed by the Neighbor-Joining method using MEGA 6 software. The numbers on the tree indicate the percentages of bootstrap support derived from 1,000 replications. The scale bar corresponds to 0.1 substitutions per nucleotide position.

BLASTn analysis of the cloned gene revealed 98.2% similarity with the alkaline protease of *B. lehensis* G1 (CP003923), 97.9% with YaB alkaline elastase subtilisin gene of *Bacillus subtilis* (M28537), and 95.4% with AprN alkaline protease of *Bacillus* sp. (AB005792). Whereas the BLASTp analysis revealed highest sequence similarity of 99.3% with both alkaline protease of *B. lehensis* MLB2 (AFK08970) and alkaline elastase YaB of *Bacillus* sp. YaB (P20724) followed by 97.8% with AprN alkaline protease of *Bacillus* sp. (BAA25184). Further, APrBL has shown 82.3 and 80.4% similarity with the subtilisin savinase of *Bacillus* sp. (P29600) and alkaline protease of *Bacillus clausii* KSM-K16 (Q99405), respectively. The APrBL protease was further compared with other proteases with respect to amino acid composition, structural features, and phylogenetic analysis. APrBL closely related to alkaline elastase YaB of *Bacillus* sp. YaB and alkaline protease BLAP of *B. lehensis* MLB2 (AFK08970). On the other hand, it distantly related to subtilisin Carlsberg (CAB56500) of *Bacillus licheniformis* and subtilisin BPN’ (P00782) from *Bacillus amyloliquefaciens* (Figure 1 and Table 1). The alkaline protease gene sequence of *B. lehensis* JO-26 (APrBL) has been submitted to GenBank (Accession No. MN104891).

**TABLE 1:** Comparison of primary structure profile of APrBL with other reported proteases including phylogenetic neighbors.

**Enzymes**	**Hydrophobic aa (%)**	**Aromatic aa (%)**	**Asp + Glu/Arg + Lys**	**Theoritical PI**	**Gravy index**	**Instability index**	**Aliphatic index**
AprBL	55.3	7.8	2.14	4.66	−0.036	26.23	85.44
YaB (P20724)	56	7.2	1.84	4.66	0.014	26.01	87.54
Subtilisin Carlsberg (X03341)	54.3	8.9	0.9	8.73	0.036	13.96	83.69
AprN (BAA25184)	55.5	7.3	1.78	4.77	−0.002	26.28	87.28
BLAP (AFK08970)	56	7.5	1.84	4.72	0.025	26.08	87.54
Subtilisin BPN′ (NC_014551)	52.6	8.6	0.89	8.73	−0.105	26.29	78.51

### Expression of the Alkaline Protease Gene in *Escherichia coli*

#### Effect of Isopropyl β-D-Thiogalactopyranoside and Temperature on Gene Expression

It is highly desirable to get the expressed protein in soluble fraction due to its easy recovery, cost-effective purification, and minimum activity loss and time consumption (Sørensen and Mortensen, 2005). Among the strategies to limit trapping of the expressed proteins in insoluble fraction and thus reducing the formation of the inclusion bodies, growth of the host organism at low temperatures with lower induction level are important (Sørensen and Mortensen, 2005; Rosano and Ceccarelli, 2014). In the present report, the growth of the host was carried out at various temperatures and different levels of induction. At 37°C, while growth of the organisms was highest, the expressed protease was largely trapped into the inclusion bodies, i.e., in insoluble fraction. On the contrary, growth at 28°C enhanced the solubility of the expressed protein despite lower growth of the host. There are several reports on the expression of alkaline protease in soluble fraction at lower temperatures of 27–30°C (Gohel and Singh, 2012; Joshi and Satyanarayana, 2013; Purohit and Singh, 2014; Devi et al., 2016).

**FIGURE 2:**
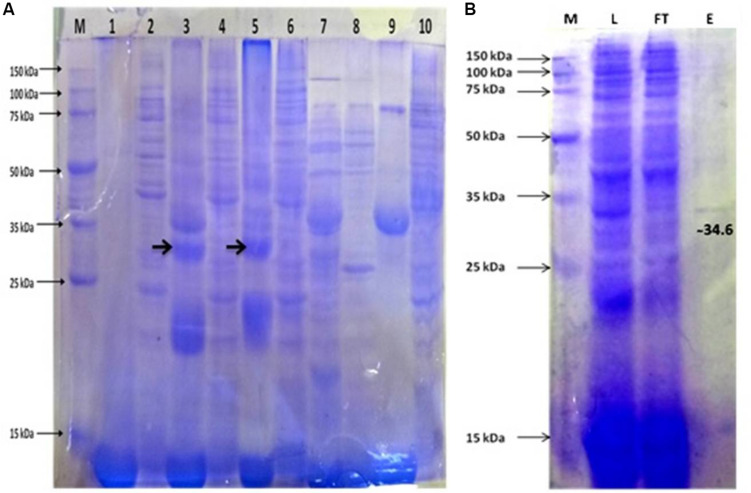
**(A)** Protein expression profile of APrBL. M: 15–150 KD, Lane 1: Preinduced soluble fraction of *Escherichia coli* BL21 (DE3) harboring recombinant APrBL-pET28a, Lane 2: Preinduced insoluble fraction of *E. coli* BL21 (DE3) harboring recombinant APrBL-pET28a, Lane 3: Soluble fraction of *E. coli* BL21 (DE3) harboring recombinant APrBL-pET28a induced with 0.2 mM IPTG, Lane 4: Insoluble fraction of *E. coli* BL21 (DE3) harboring recombinant APrBL-pET28a induced with 0.2 mM IPTG, Lane 5: Soluble fraction of *E. coli* BL21 (DE3) harboring recombinant APrBL-pET28a induced with 1 mM IPTG, Lane 6: Insoluble fraction of *E. coli* BL21 (DE3) harboring recombinant APrBL-pET28a induced with 1 mM IPTG, Lane 7: Soluble fraction of *E. coli* BL21 (DE3), Lane 8: Insoluble fraction of *E. coli* BL21 (DE3), Lane 9: Uninduced soluble fraction of *E. coli* BL21 (DE3) harboring recombinant APrBL-pET28a, Lane 10: Uninduced insoluble fraction of *E. coli* BL21 (DE3) harboring recombinant APrBL-pET28a. **(B)** Protein purification using Ni-NTA column after expression of the protein in pET28a, 28°C, 0.2 mM IPTG. M: 15–150 KD, L: Soluble fraction of *E. coli* BL21 (DE3) harboring recombinant APrBL-pET28a induced with 0.2 mM IPTG, FT: Flow through from the column, E: Column elution in 250 mM imidazole.

The synergistic effect of IPTG induction and growth temperatures on APrBL expression was reflected by the fact that despite reduced growth of the recombinant *E. coli* at 1 mM IPTG, the expression level was quite comparable at both 0.2 mM and 1 mM IPTG concentrations at both 28°C and 37°C temperatures (Figure 2A).

Although expression was higher at 1 mM IPTG induction, considering the correlation of the growth and expression level, IPTG induction at 0.2 mM can be considered optimum for the APrBL expression. Similarly, a study on the expression of cellobiose phosphorylase from *cellvibrio gilvus* revealed that enzyme activity was higher in soluble fraction when cells were grown at 25°C as compared with 37°C (Singh et al., 2010). Furthermore, the enzyme activity was higher in soluble fraction at 0.1 mM IPTG induction as compared to 1 mM IPTG (Singh et al., 2010).

#### Effect of Salt Concentration

**FIGURE 3:**
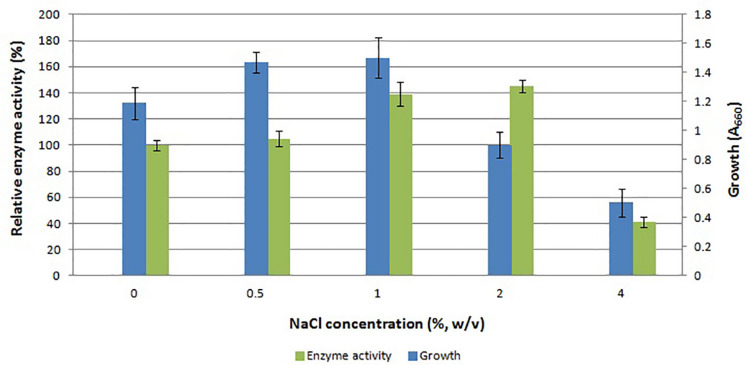
Effect of NaCl concentration on growth and APrBL activity.

There is scarce information on the effect of salt on the gene expression in *E. coli*. The main reason to study the effect of salt on the expression of APrBL protease gene was the haloalkaliphilic nature of the native strain *B. lehensis* JO-26. The maximum expression was achieved with 2% NaCl (w/v). Growth of *E. coli* along with protease expression increased up to 1% NaCl (w/v) (Figure 3). Despite reduced growth, the enzyme production was higher at 2% NaCl (w/v) concentration. At 4% NaCl (w/v) concentration, both growth of the host and protease production decreased. The data clearly suggested that with increasing salt concentration, expression of functional recombinant APrBL increased up to 2% NaCl (w/v) (Figure 3 and Supplementary Figure S1).

#### Purification of the Recombinant Protease

The recombinant protease appeared to be located in the cytoplasmic space as extracellular fraction did not contain any protease activity. The maximum expressed enzyme in soluble fraction was observed at 0.2 mM IPTG induction after 20 h of growth at 28°C and 200 rpm agitation. The soluble fraction of APrBL was purified onto Ni-NTA matrix using 250 mM imidazole for elution. The purified APrBL resolved as a single band of 34.6 ± 1.0 kDa on SDS–PAGE (Figure 2B). The purified recombinant enzyme exhibited a 16.93-fold increase in the specific activity with a yield of 84.6% (Table 2). The molecular masses of microbial alkaline proteases with some exceptions range between 15 and 40 kDa (Dodia et al., 2008; Haddar et al., 2009; Sinha and Khare, 2013; Raval et al., 2014). Alkaline proteases of similar molecular mass to APrBL have been cloned into mesophilic hosts, such as 34 kDa from *Bacillus pumilus* CBS (Jaouadi et al., 2008), 34.4 kDa from *Bacillus vallismortis* (Cheng et al., 2015), and 35.6 kDa from *Planococcus* sp. (Zhang et al., 2016). Zone of the utilization of the substrate was observed in the plate diffusion assay of the purified APrBL, confirming the activity of the enzyme (Supplementary Figure S2).

**TABLE 2:** Purification of the recombinant alkaline protease APrBL by Ni-NTA affinity chromatography.

**Enzyme preparation**	**Total activity (U)**	**Total protein (mg)**	**Specific activity (U/mg)**	**Purification fold**	**Yield (%)**
Recombinant crude fraction	1,392	4.8	290	1	100
Ni-NTA affinity Chromatography Purified fraction	1,179	0.24	4,912	16.93	84.6

#### Kinetic Parameters: *K*_m_, *V*_max_, and *K*_cat_

*K*_m_ and *V*_max_ were computed as 1.38 mg/ml and 27.14 μmol mg^–1^ min^–1^ (212.76 U/ml), respectively. The *K*_m_ is inversely proportional to the enzyme’s affinity with the substrate. The APrBL protease has low *K*_m_ value compared to other reported proteases from *Bacillus* sp. (1.53–2.5 mg/ml), metagenomic alkaline protease (1.70 mg/ml), *Bacillus halotolerance* (10 mg/ml), *Bacillus marismortui* (2.5 mg/ml), *Shewanella arctica* (1.75 mg/ml), *Idiomarina* sp. (3.76 mg/ml) (Patel et al., 2006; Dodia et al., 2008; Sinha and Khare, 2013; Qoura et al., 2015; Devi et al., 2016; Dorra et al., 2018; Zhou et al., 2018). *V*_max_ is the catalytic activity of an enzyme usually desired as high as possible. The studied enzyme had higher *V*_max_ than other reported proteases from *B. vallismortis* (49.8 μg ml^–1^min^–1^), *Bacillus circulans* (1.8 μmol/min), *B. lehensis* (25 nmol mg^–1^ s^–1^), and metagenomic protease (278.2 U/mg/min) (Joshi and Satyanarayana, 2013; Cheng et al., 2015; Devi et al., 2016; Patil et al., 2016). The results signify high affinity and catalytic efficiency of APrBL compared to other proteases.

*K*_cat_ is the maximum number of conversions of the substrate molecules per second by a single catalytic site at a given enzyme concentration (Mechri et al., 2017). *K*_cat_ is a constant independent of the amount of the enzyme. With respect to the present enzyme, the catalytic constant *K*_cat_ was deduced as 0.549 S^–1^. In comparison, Cheng et al. (2015) reported a *K*_cat_ value of 4.35 min^–1^ for an alkaline protease from *B. vallismortis*. *K*_cat_ values of 0.13 S^–1^ and 3.99 × 10^–2^S^–1^ were reported for the alkaline proteases of *Bacillus* sp. and *Bacillus pseudofirmus*, respectively (Sinha and Khare, 2013; Raval et al., 2014). However, a higher *K*_cat_ value of 289 S^–1^ has also been earlier reported with low *V*_max_ (Joshi and Satyanarayana, 2013). Overall, the kinetic parameters; *K*_m_, *V*_max_, and *K*_cat_ of the APrBL suggest its potential and suitability as a biocatalyst in comparison to the previously reported alkaline proteases.

### Characterization of Recombinant Protease

#### Effect of pH on the Enzyme Activity and Stability

**FIGURE 4:**
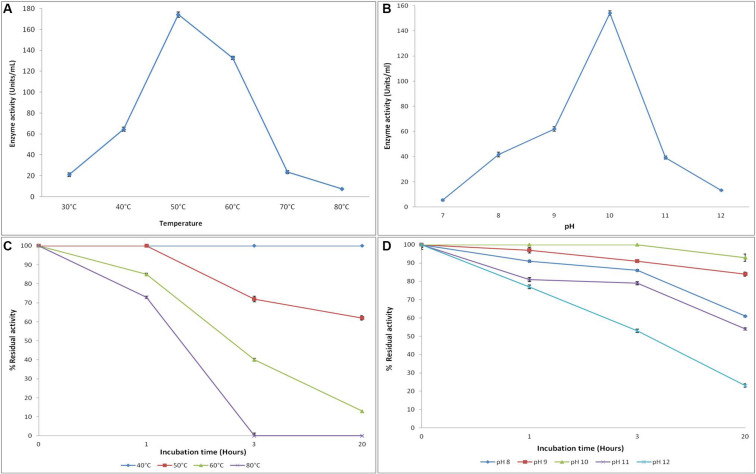
Effect of temperature and pH on the activity **(A,B)** and stability **(C,D)** of recombinant alkaline protease APrBL, respectively.

The APrBL was active in a broad range of pH 8–12 with an optimum at pH 10, thus confirming the alkaline nature of the enzyme (Figure 4B). The trend of the pH optima is consistent with the proteases of *Bacillus* sp. (Patel et al., 2006), *B. pumilus* (Jaouadi et al., 2008), haloalkaliphilic bacterium strain AH-6 (Dodia et al., 2008), *Nocardiopsis alba* (Gohel and Singh, 2012), *Bacillus* sp. (Jain et al., 2012), and *B. pseudofirmus* (Raval et al., 2014). The trends, however, vary from the pH optima of the alkaline proteases of *B. vallismortis* (optimum pH, 6.5) (Cheng et al., 2015), *Bacillus mojavensis* (optimum pH, 8.5) (Haddar et al., 2009), *Bacillus halotolerans* (optimum pH, 9) (Dorra et al., 2018), *Bacillus* sp. (optimum pH, 9) (Sinha and Khare, 2013), and *Bacillus* sp. (optimum pH, 9) (Briki et al., 2016).

The recombinant protease APrBL was highly stable in the range of pH 8–12 (Figure 4D). The enzyme was stable at pH 8–12, for 3 h, with significant stability displayed even after prolonged incubation up to 24 h at pH 9–10. The enzyme retained more than 80% of the residual activity at pH 9 and 10, while it retained 50–60% activity at pH 8. The trends, however, differ from the alkaline protease of *B. vallismortis* which was stable at pH 5.6–9.6 (Cheng et al., 2015). Effective functioning of the protease over a broad pH range is an important criterion for the application as detergent additives. The APrBL protease fulfills this condition as it was active and stable in the pH range of 8–12. The APrBL appears more efficient at alkaline pH as compared to the commercial detergent enzyme Alcalase (Novozymes A/S) produced by *B. licheniformis*, having a maximal activity at pH 8–9 (Van Kampen and Merget, 2002). The enzymatic features are quite comparable with Purafect (Genencor International Inc., United States), a genetically engineered donor *B. lentus* expressed in *Bacillus* sp. having the optimum activity at pH 10 (Van Kampen and Merget, 2002) and Maxatase (Gist-Brocades), produced by *B. licheniformis* with a maximum activity at pH 9–10 (Beg and Gupta, 2003).

#### Effect of Temperature on the Activity and Stability

The enzyme was active at temperatures 30–70°C with optimum activity at 50°C (Figure 4A). The enzyme retained 76% of the residual activity at 60°C, whereas at 40°C, 37% of the activity was evident. A sharp increase in the activity was observed from 40 to 50°C, while the activity decreased from 50 to 60°C, clearly suggesting the thermally active nature of the enzyme. These results, however, deviate from the alkaline proteases of *Planococcus* sp. (optima at 35°C) and *Bacillus* sp. (optima at 37°C) (Briki et al., 2016; Zhang et al., 2016). Interestingly, the enzyme also differs from its phylogenetic relative, BLAP alkaline protease, of the same species *B. lehensis* (Joshi and Satyanarayana, 2013). Despite sharing a common temperature optima of 50°C, while BLAP rapidly loses activity at 60°C, the APrBL retains 76% of the residual activity at this temperature.

The APrBL is stable in the range of 40°C–80°C (Figure 4C). The protease was highly stable at 40°C even after 20 h. While at 50°C, it was highly stable up to 1 h, the enzyme retained 72 and 62% of the residual activities after 3 and 20 h of incubation, respectively. At 60 and 80°C, 85 and 73% of the residual activities were evident up to 3 h, respectively, whereas 40% of the activity was retained at 60°C, and total loss of the activity was observed at 80°C after 3 h of incubation (Figure 4C). The stability profile of APrBL suggests its superiority when compared to other alkaline proteases (Haddar et al., 2009; Jellouli et al., 2011; Cheng et al., 2015).

#### Effect of Metal Ions, Surfactants, Inhibitors, and Solvents

Among the cations, while Ca^2+^ slightly stimulated the APrBL activity, Hg^2+^ completely inhibited the activity (Table 3). The trends corresponded with some earlier reports on alkaline proteases (Patel et al., 2006; Cheng et al., 2015). Hg^2+^ reacts with the aromatic ring of tryptophan oxidizing its indole ring (Liu et al., 2010). APrBL activity was marginally inhibited by Mg^2+^, Mn^2+^, Zn^2+^, and Co^2+^, while Fe^2+^ significantly reduced the activity by 60% (Table 3). In a similar manner, an alkaline protease from *B. halotolerans* was marginally inhibited by Mg^2+^, Zn^2+^, and Co^2+^ while strongly inhibited by Fe^2+^ (Dorra et al., 2018). In comparison, Zn^2+^ inhibited the activity by 50%, while Fe^2+^ marginally inhibited the activity (Devi et al., 2016). Majority of the proteases of *Bacillus* sp. are induced by Ca^2+^ (Dodia et al., 2008; Haddar et al., 2009). Ca^2+^ marginally stimulated the activity of alkaline protease as also reported earlier for serine proteases of *Bacillus* sp. (Jain et al., 2012). Despite some benefits, calcium dependency raises limitations of using enzymes in detergents containing chelating agents (Sato et al., 1990). Calcium-independent protease variants can be obtained by removing the calcium-binding loop using directed mutagenesis (Strausberg et al., 1995). Therefore, the calcium-independent character of the APrBL adds to its suitability as a detergent additive.

**TABLE 3:** Effect of various additives on the purified APrBL enzyme activity.

**Additives**	**Residual activity (%)**
**Solvents [10% (v/v)]**	
Control	100 ± 1
n-Hexane	85.39 ± 3.8
DMF	35.35 ± 3.07
Propanol	75.36 ± 1.55
Chloroform	378.51 ± 1.55
Glycerol	22.27 ± 0.69
Toluene	113.07 ± 0.78
n-Butanol	118.2 ± 2.84
Benzene	146.95 ± 3.89
Methanol	76.15 ± 0.79
Ethanol	53.83 ± 1.52
**Metal ions (5 mM)**	
Control	100 ± 1
Ca^2+^	107.8 ± 4.26
Mg^2+^	77.7 ± 2.55
Zn^2+^	63.2 ± 2.55
Mn^2+^	76.4 ± 4.24
Hg^2+^	0
Co^2+^	84.3 ± 1.70
Fe^2+^	38.5 ± 3.40
**Surfactants/inhibitors**	
Control	100 ± 1
EDTA (5 mM)	46.15 ± 2.17
PMSF (5 mM)	0
DTT (5 mM)	36.92 ± 4.35
β-ME (5 mM)	30.765 ± 2.17
SDS [1% (w/v)]	275.38 ± 4.35
Triton X-100 (1%)	36.92 ± 2.17
Tween 80 [1% (v/v)]	54.61 ± 5.44

Various inhibitors were assessed for their effect on the protease activity. The activity was completely inhibited by PMSF, suggesting its serine nature (Table 3). The essential serine residue in the active site is sulfonated by PMSF, leading to the total loss of the activity (Mechri et al., 2017). Further, APrBL was inhibited by DTT, β-ME, and EDTA, retaining 37, 31, and 66% activities, respectively (Table 3).

Among the surfactants, SDS stimulated APrBL activity by 2.35-fold (Table 3). Previously, 1.55- and 3.35-fold enhancement in the activity are reported by SDS in *Nocardiopsis alba* and *Bacillus* sp. (Gohel and Singh, 2012; Jain et al., 2012). APrBL enzyme was relatively less stable in non-ionic surfactants such as Triton X-100 and Tween-80 as compared to some earlier reported proteases (Annamalai et al., 2014). Recently, a 50% induction of an alkaline protease activity by SDS was reported (Dorra et al., 2018). However, majority of the serine alkaline protease are reported as SDS labile (Haddar et al., 2009; Jellouli et al., 2011; Raval et al., 2014; Zhang et al., 2016). Due to the amphiphilic nature, SDS reacts with the amino acids, leading to protein unfolding and loss of activity. On the contrary, SDS favorably changes the confirmation of the APrBL protease and stimulates the activity. The recombinant protease might be slightly misfolded yet retains full activity after the interaction with SDS.

The alkaline proteases have a potential role in ester and peptide synthesis under non-aqueous conditions (Jellouli et al., 2011). The advantages of non-aqueous catalysis include the use of water-insoluble substrates, reduced side reactions, reduced microbial contamination, easy product separation and recovery, and thermodynamic equilibrium favoring synthesis (Klibanov, 2001). However, enzymes lose their conformation in organic solvents, leading to the loss of structural flexibility due to the stripping off of the essential water layer (Doukyu and Ogino, 2010). Hence, enzyme activity and stability in organic solvents become major challenge. APrBL activity was significantly enhanced by chloroform and benzene and moderately enhanced by n-butanol and toluene (Table 3). On the contrary, adverse effect on the activity of alkaline protease by benzene and toluene has been reported earlier (Joshi and Satyanarayana, 2013). Further, methanol, ethanol, and isopropanol positively affected the APrBL activity, as against the toluene and hexane, which considerably acted against the enzyme (Devi et al., 2016). In the present study, dimethylformamide (DMF) and glycerol significantly inhibited the APrBL activity, while the activity was moderately inhibited by n-hexane, propanol, methanol, and ethanol (Table 3). There are several studies on the stimulation of protease activity by methanol and ethanol (Thumar and Singh, 2009; Sinha and Khare, 2014; Mechri et al., 2017). APrBL enzyme was studied in a broad range of organic solvents of variable log *P*-value (−2.32 to 3.9). Solvents with logP < 4.0 are considered as extremely toxic due to their greater partitioning into the aqueous and hydrophobic layers (Mechri et al., 2017). Thus, due to the disruption of the hydrogen bonds and hydrophobic interactions depriving the water hydration shell of the protein, the structural flexibility and functionality are lost (Barberis et al., 2006; Mechri et al., 2017). The haloalkaliphilic bacteria decrease the water activity in the cytoplasm by accumulating salt and thus mimic a non-aqueous system. The enzymes by adapting to high salt are able to function in non-aqueous media. The APrBL was stable in all solvents except those of log *P* < 0.24, which might be due to a larger number of acidic amino acids on its surface as evident from its structure (Table 1). The negative charges enhance protein solubility through hydrated ion network with cations or by preventing the protein aggregation *via* electrostatic repulsion on the protein surface (Jain et al., 2012). The experiments were carried out in triplicates, and the results are presented as the mean values.

#### Structure–Function Analysis of APrBL

The physicochemical properties of APrBL elucidated by ProtParam, including the amino acid composition, instability index, theoretical pI, molecular weight, grand average of hydropathicity (GRAVY), aliphatic index, and total number of negatively and positively charged residues, are shown in Table 3. Proteins with an instability index of below 40 are predicted stable, while those with values above 40 are predicted unstable (Guruprasad et al., 1990). Instability index was as low as 26.23, clearly suggesting the stability of the protein. Further, aliphatic index and grand average of hydropathicity (GRAVY) index were 85.44 and −0.036, respectively. The GRAVY index is the sum of hydropathy values of all the amino acids divided by the number of residues in the sequence. A low GRAVY index of APrBL indicates low hydrophobicity and high hydrophilicity of the protein, suggesting a better interaction with water. The aliphatic index is measured by the relative volume occupied by the aliphatic side chains. It may enhance the thermostability of the globular proteins (Ikai, 1980). A high aliphatic index of the protein indicates its enhanced thermostability over a broad range of temperatures, as reflected by the APrBL and supported by the experimental data. A high number of negatively charged amino acids (Asp + Glu: 30) as compared to positively charged amino acids (Arg + Lys: 14) is believed to confer alkali-halo stability of the protease. The ratio of Asp + Glu/Arg + Lys was found to be 2.14, which is highest for APrBL in comparison with other phylogenetic relatives (Table 1). Normally, in the presence of salt ions, the enzyme gets precipitated. However, with respect to the halophilic enzymes, negative charges stabilize water and/or ion-binding essential for the tertiary or quaternary structure (Takenaka et al., 2011). The negative charges promote protein refolding and prevent aggregation. The APrBL exhibited 8.90% negatively charged residues, which is higher than other alkaline proteases, M-protease (3.36%), subtilisin BPN’ (5.08%), and subtilisin Carlsberg (7.38%) (Jacobs et al., 1985; Gallagher et al., 1996; Shirai et al., 1997). The features of the primary structure of the APrBL align with some earlier reported proteases: BLAP protease (Joshi and Satyanarayana, 2013), YaB protease (Kaneko et al., 1989), and AprN (Masui et al., 1998) (Table 1). On the contrary, non-halophilic subtilisin Carlsberg and subtilisin BPN’ have a reduced number of negatively charged and hydrophobic residues, high number of aromatic amino acids, high GRAVY index, and low aliphatic index. Moreover, APrBL exhibited as high as 55.3% hydrophobic amino acids which might be responsible for solvent stability (Karan et al., 2011). Solvent stability of the enzyme was also supported by the hydropathy profile of the amino acid sequence of APrBL, which reveals hydrophobic nature of the protein (Supplementary Figure S3). Interestingly, although high glycine and less proline content is a characteristic feature of the cold-adapted enzymes, it was observed in thermo active APrBL, which suggests that this feature may not be solely responsible for thermal/cold adaptation.

#### Secondary and Three-Dimensional Structure Modeling of the APrBL

**FIGURE 5:**
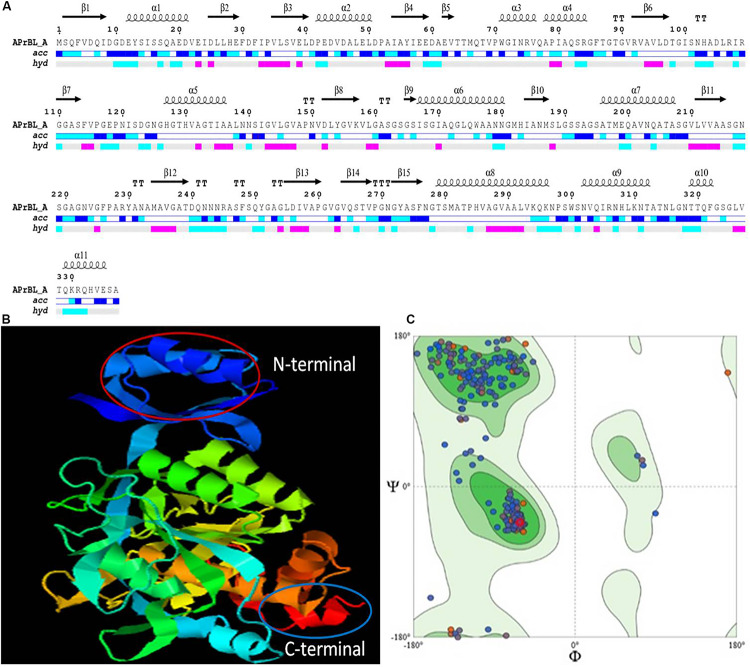
**(A)** Secondary structure prediction of APrBL by ENDscript 2.0. On the top are secondary structures (squiggles: helices, arrows: beta strands, TT letters: beta turns). On the bottom, ***acc*** denotes bar of accessibility (dark blue: accessible, light blue: intermediate, white: buried) and ***hyd*** denotes a bar of hydropathy (pink: hydrophobic, light blue: hydrophilic, white neutral). **(B)** Three-dimensional (3D) structure showing N-terminal and C-terminal of APrBL generated using I-TASSER server. **(C)** Ramachandran plot for the predicted 3D structure of APrBL by SWISS-MODEL. A total of 95.22% of the residues are distributed in the most favored region which predicts its stability.

Secondary structure of APrBL as analyzed by END script 2.0 indicated 10 α-helices and 16 β-sheets (Figure 5A). The SOPMA tool revealed that the APrBL contained 31.75% α-helices, 22.55% β-strands, and 45.70% coils, suggesting the dominance of the coils. The halophilic and alkaliphilic enzymes need to maintain a balance between enough rigidity to prevent the unfolding and flexibility to allow the motions necessary for the catalysis (Zorgani et al., 2014). Therefore, the coil regions in APrBL provide a flexible conformation enhancing its activity and stability under the saline and alkaline conditions. On the contrary, non-halophilic enzymes contain more α-helix and β-strand-forming regions (Zorgani et al., 2014). A similar trend was observed in an alkaline protease from *B. pumilus* (Baweja et al., 2016). Further, low K_m_ and high K_cat_ values of APrBL could be attributed to the dominance of coils which confers structural flexibility to the enzyme. Structural flexibility in the active site of the enzyme might facilitate efficient substrate binding and catalysis. Nevertheless, stability of APrBL at high temperatures and alkaline pH, solvents, and detergent is presumably due to the α-helices and β-strands that provide rigidity to the structure (Fields et al., 2015).

It is hypothesized that a high number of hydrophobic amino acids facilitated APrBL folding in a way to generate more grooves to avoid water molecule. High hydrophobicity in protein promotes stability in stressful conditions. However, the balance of the hydrophobic and hydrophilic regions is important for the interaction of the protein with its surrounding medium, at the same time to increase the substrate binding capacity in the core region required for the efficient catalysis (Panja et al., 2020).

The tertiary structure model was generated by I-TASSER which combines the methods of threading, *ab initio* modeling, and structural refinement (Figure 5B). The 3D model explained that amino acids forming catalytic triad are in close proximity in tertiary structure but located far apart in primary structure. Therefore, appropriate folding of the enzyme brings amino acids together which are crucial to the catalytic activity in the tertiary structure of the enzyme. The structure had a good C score of 1.56 (range −5 to 2) and TM score of 0.93. A TM score of > 0.5 indicates a model of correct topology. The quality of 3D structural model was validated by structure analysis tool of SWISS-MODEL. High score value for local quality plot (> 0.6) supports the structure of the APrBL as a good feature. In addition, Ramachandran plot indicated that 95.22% of the amino acid residues are located in the most favored region, which further validates as a good quality model (Figure 5C).

#### Homology Modeling Analysis of APrBL

SWISS-MODEL workspace of expasy platform was used to search template for homology modeling. Homology search of APrBL has shown highest structural similarity of 80.97% with the crystal structure of alkaline M-protease (1wsd.1.A protein) from *Bacillus clausii* strain KSM-K16 (PubMed ID: 9278275). An alkaline M-protease optimally acts at pH 12.3 (Shirai et al., 1997). In homology modeling, QMEAN, a composite estimator, provides both global (i.e., for the entire structure) and local (i.e., per residue) quality estimates based on the single model (swissmodel.expasy.org). The QMEAN Z-score provides an estimate of the “degree of nativeness” of the structure which indicates whether this model would fit into the experimental structures of similar size (swissmodel.expasy.org). QMEAN *Z*-score near zero suggests correspondence between the model and the experimental structure. Lower scores are indicative of low quality of the model. A QMEAN *Z*-score of −0.12 for the APrBL supports the homology model.

### Applications of the Recombinant Alkaline Protease APrBL

#### Wash Performance Analysis of APrBL

**FIGURE 6:**
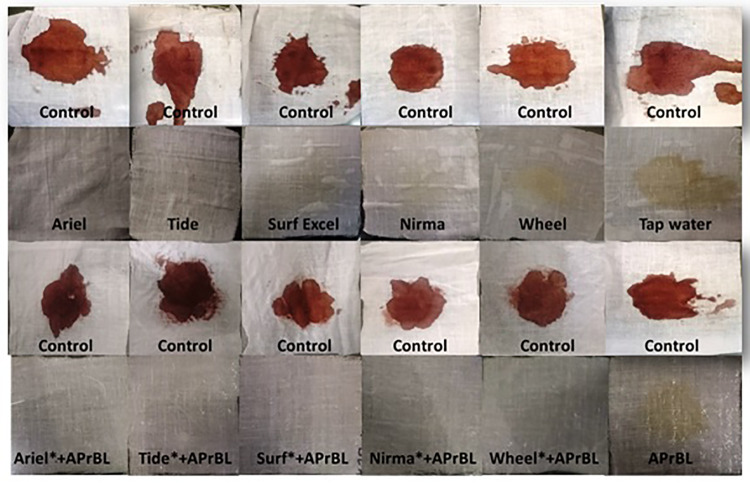
Wash performance analysis showing different washing conditions for bloodstain removal.

Stain removal ability of the APrBL was analyzed on cotton cloths. The washing test of the APrBL revealed significant removal of bloodstain from the cotton cloth when compared to controls (Figure 6). The washing efficiency of the detergents alone revealed that a complete stain removal with Ariel and Tide, while the detergents Surf, Nirma, and Wheel could not completely remove the stain. However, the stain was completely removed with the supplementation of the APrBL in the detergents (Figure 6). During the removal of the proteinaceous substrates in the cloths, the proteins are degraded into smaller peptides due to the action of the detergent and/or supplemented proteases. In case of the smaller peptides, they are either solubilized into the washing solution or get deposited back on the fabric. Therefore, the suitable enzymes with improved hydrolysis would aid the stain removal and prevent redeposition. In this investigation, the treatment with APrBL plus detergent not only removed the stain by hydrolyzing the protein but also prevented protein redeposition on the fabric. The application of alkaline proteases in bloodstain removal is described in the literature (Beg and Gupta, 2003; Jaouadi et al., 2008; Patil et al., 2016; Mechri et al., 2017; Rekik et al., 2019). Noticeably, APrBL efficiently removed the bloodstains within 10 min compared to 30–60 min by the previously reported proteases (Beg and Gupta, 2003; Jaouadi et al., 2008; Patil et al., 2016; Mechri et al., 2017; Rekik et al., 2019). These findings further add to the potential of APrBL in detergent industry.

#### Whey Protein Hydrolysis

Whey, a by-product of the dairy industry, provides important nutritional ingredients (Sinha et al., 2007). The most important proteins in whey are α-lactalbumin and β-lactoglobulin, representing 70–80% of its total protein content (Smithers et al., 1996). Besides, whey possesses antioxidant properties attributed to high presence of sulfur-containing amino acids. However, due to its high organic content, it is also considered as one of the most polluting agents of water bodies (Moreno-Indias et al., 2009).

**FIGURE 7:**
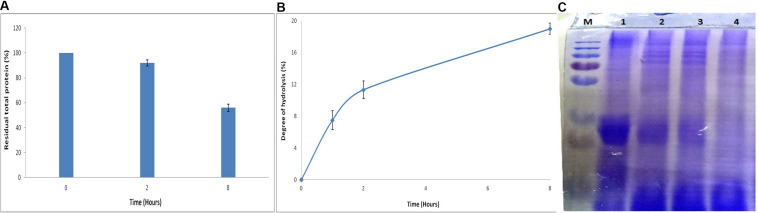
**(A)** Effect of APrBL enzyme on total protein in milk whey. **(B)** Effect of APrBL enzyme on degree of hydrolysis of milk whey. **(C)** Sodium dodecyl sulfate–polyacrylamide gel electrophoresis (SDS–PAGE) profile of untreated and enzymatically hydrolyzed milk whey proteins by APrBL enzyme. M: Marker; Lane 1: untreated whey; Lane 2: treated, 1 h; Lane 3: treated, 2 h; Lane 4: treated, 8 h.

Effect of APrBL on whey protein led to a decreased total protein content (Figure 7A). Hydrolysis of the whey protein results into small peptides of varied size and free amino acids. SDS–PAGE profile reveals action of APrBL in the gradual conversion of high-molecular-weight whey proteins into low-molecular-weight smaller peptides (Figure 7C). The protein hydrolysates exhibit antimicrobial, antihypertensive, antioxidant, and lipid-lowering properties (Silvestre et al., 2013). Hydrolyzed whey protein-based formulas are favored for infants intolerant to cow’s milk protein (Sinha et al., 2007). In our study, 19% of the hydrolysis was achieved in 8 h by APrBL recombinant protease (Figure 7B). In majority of the cases, the DH for whey proteins varies in the range of 5–23% (Sinha and Khare, 2014). A 28 and 35% hydrolysis of whey protein was obtained by free and immobilized protease, respectively (Sinha and Khare, 2014). The protein hydrolysates provide growth-supportive peptides, eliminating the need of expensive nutrients in the growth medium (Pintado et al., 1999). The enzymatic processes provide environment-friendly processing conditions with fewer undesirable effects as compared to other conventional methods.

## Conclusion

We successfully cloned an alkaline protease gene (APrBL) from *B. lehensis* JO-26 and optimally expressed in *E. coli* BL21 (DE3). The effect of NaCl on the expression of the alkaline protease was demonstrated. Maximum enzyme expression was achieved with 0.2 mM IPTG, 2% NaCl (w/v), and 28°C growth temperature. The APrBL has shown high activity and excellent stability over a wide range of pH and temperatures, with the tolerance against surfactants and reducing agents. Kinetic parameters *K*_m_, *V*_max_, and *K*_cat_ of the APrBL protease suggest its potential as a biocatalyst. Structural features of APrBL corroborate the experimental data. It was also evident that high glycine and reduced proline content are not solely responsible for the thermal/cold adaptation. The enzyme can also be used in peptide synthesis under non-aqueous environment due to its stability and activity in organic solvents. Applications of this enzyme as a detergent supplement and in whey protein hydrolysis clearly suggest its suitability in detergent and food industries. We understand this is the first report on the cloning, expression, characterization, and structural elucidation of an alkaline serine protease from a saline desert.

## Data Availability Statement

The alkaline protease gene sequence of Bacillus lehensis JO-26 (APrBL) has been submitted to GenBank (Accession No. MN104891).

## Author Contributions

HB and SS conceived and designed the experiments. HB performed the experiments, analyzed data and wrote the manuscript. SS helped in analysis and interpretation of the data. SS supervised the work.

## Conflict of Interest

The authors declare that the research was conducted in the absence of any commercial or financial relationships that could be construed as a potential conflict of interest.

## Footnotes

1 http://web.expasy.org/protparam/

2 https://prosite.expasy.org/

3 http://swissmodel.expasy.org/workspace
