# Supplementary figures and images for: Cloning, Expression, and Structural Elucidation of a Biotechnologically Potential Alkaline Serine Protease From a Newly Isolated Haloalkaliphilic Bacillus lehensis JO-26
Source: Front Microbiol. 2020 Jun 3;11:941. doi: 10.3389/fmicb.2020.00941 (PMC7283590; doi:10.3389/fmicb.2020.00941)

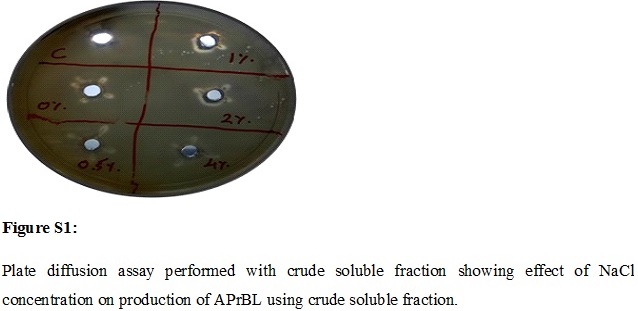

Supplement: Supplementary file 1 [file Image_1.JPEG]

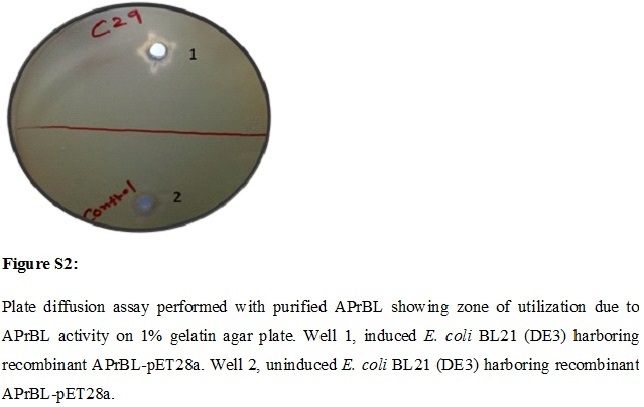

Supplement: Supplementary file 2 [file Image_2.JPEG]

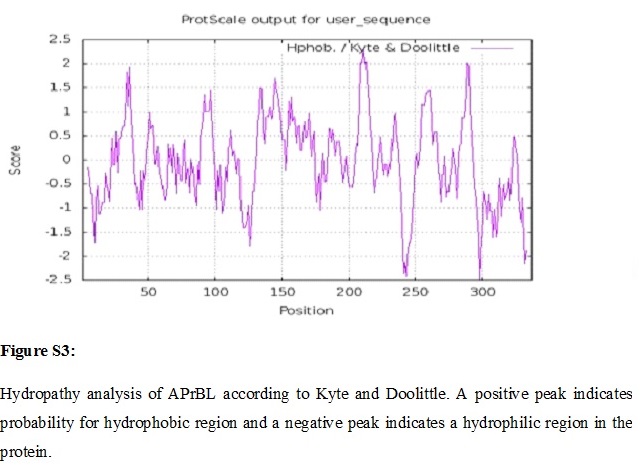

Supplement: Supplementary file 3 [file Image_3.JPEG]
